# A Combination of Flavonoids Suppresses Cell Proliferation and the E6 Oncogenic Pathway in Human Papillomavirus-Transformed Cells

**DOI:** 10.3390/pathogens14030221

**Published:** 2025-02-24

**Authors:** Federico De Marco, Fabio Altieri, Stefano Giuliani, Italia Falcone, Susanna Falcucci, Mariassunta Tedesco, Roberto Becelli

**Affiliations:** 1 IRCCS Regina Elena National Cancer Institute, 00144 Rome, Italy; stefano.giuliani@ifo.it (S.G.); italia.falcone@ifo.it (I.F.); Susanna.falcucci@iss.it (S.F.); 2 Medicine and Pharmacy Faculty, Sapienza Rome University, Ple Aldo Moro 5, 00185 Rome, Italy; fabio.altieri@uniroma1.it (F.A.); mariassunta.tedesco@uniroma1.it (M.T.); 3 Medicine and Psychology Faculty, Sapienza Rome University, Via di Grottarossa 1035, 00189 Rome, Italy

**Keywords:** kaempferol, galangin, luteolin, chrysin, quercetin, apigenin, E6, anti-clonal effect, flavonoids

## Abstract

Despite the availability of excellent HPV-specific vaccines, HPV-related conditions and, notably, their related neoplastic diseases are expected to impact human health for many years to come. Polyphenols and flavonoids are a large class of natural products, credited with a wide range of pharmacological properties including antineoplastic activity. However, the currently available data depict a rather heterogeneous and sometimes contradictory landscape, and no univocal conclusions can be drawn. To shed light on such a controversial issue, a restricted list of promising polyphenols were evaluated for their antineoplastic activity on HPV-transformed cells. Among them, Kaempferol, Galangin, and Luteolin proved to have distinct anti-clonal activity with ID_50_ values, respectively, of 1.25, 6.25, and 3.0 microMolar, and three other compounds, namely, Chrysin, Quercetin, and Apigenin, showed fair although less intense activity with ID values, respectively, of 25.0, 40, and 25 microMolar. Interestingly, a distinct anti-proliferative effect could also be suggested for Kaempferol, Luteolin, and Apigenine. Cooperative anti-clonal effects could be suggested for binary and ternary compositions made of Kaepferol, Galangin, and Luteolin once combined at concentrations ranging from 2 to 8 microMolar. At these concentrations, the single components and the triple combination induced distinct cell cycle modulation associated with marked restoration of the p53 and p21Cip1/Waf1 levels, consistent with the disruption of the E6/E6AP interaction whose continuous activity is necessary for both the induction and maintenance of the viral-induced neoplastic phenotype.

## 1. Introduction

Human Papillomaviruses (HPVs) are a large family of small DNA viruses accounting for over 200 different types capable of infecting human skin or mucosal epithelia and which are mostly transmitted by direct contact. Typically, infections are either completely unapparent or mildly/minimally symptomatic, causing small proliferative lesions that persist for months and are ultimately spontaneously cleared [1].

HPV infection of the cervico-vaginal epithelia is a very common occurrence, estimated to affect all women at least once in their lives [2]. Once these lesions are sustained by a few types of the *AlphaPapillomavirus* genus, the so-called High-Risk HPV (HR-HPV), moderate to severe dysplastic lesions can be generated. These lesions tend to persist and may eventually give rise to invasive cervical cancer [3]. Although cancer is indeed a rare complication of viral infection, the very high prevalence of infection makes it a rather common occurrence. Indeed, cervical cancer is the third most common cause of neoplastic death for women at the global level, and the eighth leading cause of neoplastic death for women in Italy and Southern Europe [4,5]. In addition to the obvious burden of personal anxiety, pain, and deaths of any kind of neoplastic disease, cervical carcinoma, because of its peak incidence in the 3rd–5th decades of life [6], affects women in the most productive and qualitative period of their lives, thus posing a very high burden on families, communities, and society [7,8]. In addition, HR-HPVs are also related, although with only partially related mechanisms, to the occurrence of certain oropharyngeal cancers, tonsillar cancers, and anorectal cancers [9].

HPV-specific vaccines were released at the beginning of the 21st century. These vaccines are effective against up to nine of the most oncogenic HPV types, have excellent safety and efficacy profiles, and have the potential to almost completely eradicate all kinds of HR-HPV-related conditions [10]. However, alongside the theoretical concern raised by the possible enhanced circulation of those HR-HPV types not covered by vaccine formulations, vaccine campaigns are dramatically failing. Indeed, because of economic, social, and cultural reasons, coverage rates are largely unsatisfactory in all regions, and these figures are steadily worsening [11]. Sadly, poor vaccine coverage mostly occurs in medium- and low-income countries and among social groups and geographical areas where HPV infections and related cancers are more prevalent [7]. Thus, over the next decades, more than 50% of the world population is expected to remain fully susceptible to several, potentially preventable, infectious and neoplastic diseases [9,12].

A second-line, non-vaccine-based tool for the prevention of HR-HPV-related conditions is provided by the early diagnosis of pre-neoplastic lesions. Indeed, after the establishment of an HR-HPV infection, a long and complex series of events is needed for the development of a fully neoplastic phenotype [13]. This process, known as neoplastic progression, usually takes years to complete and is accompanied, at least in the case of cervical cancer, by a distinct pattern of progressively severe cyto-histological signs. Based on these signs, the early detection of pre-neoplastic lesions can be easily accomplished largely before the beginning of invasive growth [9].

However, once a dysplastic cervical lesion has been detected, it must be surgically removed. A few different technical approaches are available to this end, mostly consisting of LASER excision, electrosurgical procedures (called Loop Electrosurgical Excision Procedures (LEEPs)) or Cold Knife Conization. Each of them is rather conservative, minimally invasive, and adequate for the day-surgery setting. Conversely, all of them need to be implemented by highly qualified, and, hence, numerically limited, surgeons. They are also expensive and technically demanding and, worst of all, all of them are burdened by a high recurrence rate so that close monitoring and follow-up is needed, often mandating repeated surgery sessions. Such a high recurrence rate is due both to the incomplete removal of the primary lesions and their secondary implantation cells due to the sampling/removal procedure [14].

Sadly, the same negative sociological and psychological attitudes toward HPV vaccination also apply to cervical screening campaigns that are undertaken by only a minority of the general population. Moreover, it must be considered that the option of the early detection of pre-neoplastic lesions is not available for the cases of anorectal, oropharyngeal, and laryngeal cancers because no specific cyto-histological marker has thus far been identified for pre-neoplastic lesions in these cases [15]. Thus, the burden of HPV-related conditions is going to persist for years at the global level and new drugs to prevent and cure established HPV infections are urgently needed.

Polyphenols are a large family of molecules, largely diffused in food, nutraceuticals, and in officinal plants. They are very popular and credited as having a wide range of pharmacological properties including antioxidant, antimicrobial, and antidiabetic, cell-protective, and antineoplastic activity [16,17,18,19]. Flavonoids are a polyphenol sub-class of plant secondary metabolic products extensively found in fruits and vegetables and reported to have a variety of immune-modulatory, anti-inflammatory, and antimicrobial functions in mammals [20,21]. A large number of scientific reports have also dealt with their potentially antineoplastic effects [17,18], although available data depict a rather heterogeneous and sometimes contradictory landscape, and no univocal conclusion can be drawn.

Here, we report our recent results showing that the well-known polyphenols/flavonoids Kaempferol, Galangin, Luteolin, Chrysin, Quercetin, and Apigenine notably display strong anti-clonal, anti-proliferative, and anti-metabolic activity on cell lines transformed by HPV16, HPV18, or HPV68. These effects are coupled with a partial arrest of the cell cycle at the G_2_/M checkpoint, with indirect evidence supporting the hypothesis that their administration can disrupt the E6-E6AP interaction, a crucial step in the E6 oncogene-mediated p53 suppression.

The combination of three of them proved more active than any single component, paving the way to the possible development of oncogene-suppressing low-molecular-weight drugs.

## 2. Materials and Methods

Chemicals

The molecules listed in Table 1 (and hereafter named RNPs for brevity) have been assayed for antineoplastic activity.

Each of them was obtained as 99% purified materials from Sigma-Aldrich Co (Merk Life Science Srl. Via Monte Rosa, 93. Milano, MI, Italy). Mother solutions of each RNP were prepared in cell-grade pure DMSO at 50 μM and stored at −20 °C in 10 μL aliquots until use.

Cells

A total of four cell lines were used for this study. HeLa cells, the first established continuous cell line, were originally derived from a HPV18-positive metastatic cervical carcinoma [22]. They harbor 10–50 transcriptionally active and variably rearranged/mutated copies of the HPV18 genome, integrated into the host genome at 4 different chromosomic loci. SiHa cells are an epithelioid-like cell line derived from an invasive cervical squamous carcinoma [23] harboring a single, transcriptionally active, partially deleted copy of the HPV16 genome. Ca-Ski cells, originally derived by Baker CC and colleagues [24] harbor multiple copies of transcriptionally active HPV16 genomes. Both HeLa and SiHa lines were a kind gift from Prof. MS Campo, at The Beatson Institute for Cancer Research, Glasgow, Scotland, UK. These two cell lines, because of their versatility and ease of use, were used for most of the experiments reported. Ca-Ski cells were originally obtained through the courtesy of Prof. Matthias Dürst, at the Deutsch Krebsforschungzentrum, Heidelberg, FRG. These cells used together with SiHa provided a model for the genetic instability and variability of cervical cancers. The ME180 cells are a cell line harboring a few transcriptionally active copies of HPV68 and provided an in vitro model for dysplastic/neoplastic lesions induced by HPV with intermediate oncogenic risk [25]. Hela, Siha, Ca-Ski, and ME180, are among the most used experimental models for HPV transformation. Indeed, as a whole, they can recapitulate most of crucial features of cervical cancers such as the plurality of the viral strain involved, the variability in random events such as viral copy number variation, site of integration, and genetic rearrangement and de-regulation and offer a rather satisfactory model for basic and translational studies [3]. They were grown in high-glucose DMEM supplemented with 10% fetal calf serum (FCS). Each cell line was sub-cultured twice a week at the appropriate split ratio according to the specific proliferative index. In no cases were antibiotics or antimycotics added to the culture media. For the present study, all cell lines were retrieved from an in-house cell archive facility.

Anti-clonal activity

The anti-clonal activity was evaluated both by the current standard qualitative plating efficiency assay and by quantitative live cell imaging analysis (INCUCYTE S3 Live Cell Analysis Imaging System-Sartorius Welwyn Garden City, Hertfordshire, UK). For qualitative plating efficiency, assay cells were seeded in a 96-well plate at 120–160 cells per well (i.e., 4–5 cell/mm^2^), a density allowing the development of roughly 50 clones per well, either in medium containing the testing molecules or in plain medium and incubated without any further manipulation. After 8–9 days of incubation, according to the cell line-specific proliferative index, the cultures were decanted and stained with 0.2% CV/methanol for 5 min at room temperature, air-dried, and visually inspected and evaluated under low magnification. For quantitative evaluation, cells were seeded and treated as above and incubated for up to 6–7 days in the above mentioned INCUCYTE S3 Imaging System, purghased from Sartorius Italy Srl. 20814 Varedo (MB), Italy. Images were acquired at 12 h intervals and colony formation and percentage of growth area covered (confluence) were then monitored through automated image analysis. The analysis parameters were as follows: segmentation adjustment = 0.8; hole fill < 7000 µm^2^; adjusted pixel = −2. Objects were scored as colonies once made up of at least 24 cells, i.e., they had an area above 24,000 µm^2^ and an eccentricity of <0.750. In both cases, any condition was assayed in four to eight replicates and the anti-clonal activity was evaluated comparing the colony-forming units (CFUs) of treated versus control culture. The minimal concentration able to reduce 50% the CFUs of treated versus control culture was defined as the 50% Colony-forming Inhibitory Dose (CID_50_).

For washout experiments, cells (HeLa or SiHa) at clonal density were plated as above. After overnight adhesion, cultures were washed twice with PBS and single RNPs or their combinations were then administered. Treatments were allowed to stay on cells for 4/8/16/24 h. After that, the media were washed out, cells again washed twice and refed with plain fresh medium and the anti-clonal effect was evaluated by live cell image analysis, as above.

Anti-proliferative activity

For anti-proliferative assay, cells were seeded in 96-well microplates at 3200 cells/per well (i.e., 100 cell/mm^2^), a density allowing exponential growth rate for the subsequent 8-day incubation. After overnight adhesion, cell monolayers were washed and replenished with fresh media with different testing molecules, plain medium served as cell growth control. Cell proliferation was evaluated by confluence time-course measured through INCUCTE S3 imaging Systems. Images were acquired at 12 h intervals. The analysis parameters were as follows: segmentation adjustment = 0.9; hole fill < 7000 µm^2^; adjusted pixel = 0.

Cell Viability

Cell viability was assessed by WST assay. Briefly, 2 × 10^4^ cells per well were seeded in a 96-well plate and allowed to adhere overnight at 37 °C in a 5% CO_2_ atmosphere. Monolayers where then washed with PBS and fed with 100 μL complete medium containing either individual RNPs or RNPs mixed at indicated concentration and incubated for a further 24 to 48 h. A volume of 10 µL of cell proliferation reagent (Cell Counting Kit-8 (CCK-8) MedChem express, DBA, Milan, MI, Italy, cat. HY-K0301) was added to each well and incubated at 37 °C under 5% CO_2_ in a humidified incubator for a further 2 h. The enzymatic reduction of WST-8[2-(2-methoxy-4-nitrophenyl)3-(4-nitrophenyl)-5-(2,4-disulfophenyl)-2H-tetrazol-ium, monosodium salt] to water-soluble formazan by cell microsomal Dehydrogenases was then used as a surrogate marker of global cell viability, comparing, in an Appliskan^®^ plate reader (Thermofisher Scientific, Monza, Italy), the A_450_ of treated cultures with that of control untreated cells.

Cell cycle analysis

Cells were seeded into 6-well plates (3.5 × 10^5^ cells/well, 3.5 × 10^4^/cm^2^) and the effect of single RNPs or their combinations was evaluated after 24 h of incubation. Propidium iodide-based staining of DNA content was used to measure the percentage of cells in each cell cycle phase (G_0_/G_1_, S, and G_2_/M). Briefly, cells were detached by trypsin/EDTA, harvested by centrifugation, washed in HBSS (Sigma Aldrich, Merk Life Science S.r.l. 20149 Milano, MI, Italy) and fixed by adding 70% cold ethanol drop-wise to the pellet under continuous mixing. After 1 h incubation at 4 °C, the cells were washed twice in HBSS buffer. RNA was degraded by adding 0.2 mg/mL RNAse (Sigma Aldrich, Merk Life Science S.r.l. 20149 Milano, MI, Italy, cat. R6513) and incubating for 15 min at 37 °C. Then, 60 μg/mL propidium iodide (Sigma Aldrich, Merk Life Science S.r.l. 20149 Milano, MI, Italy, cat. P4170) was added and cells were incubated for a further 45 min at 37 °C in the dark. Before flow cytometry analysis, samples were centrifuged and resuspended in HBSS. Samples were analyzed using a BD FACSCantoTM flow cytometer (BD Biosciences, Milan, MI, Italy).

Immunoblotting analysis

For immunoblotting assay, 7.5 × 10^5^ cells were plated in 60 mm diameter dishes (roughly 35 × 10^3^ cells/cm^2^ ). After 24 h, incubation media were discarded and replaced with fresh media completed with compounds and incubated overnight. Monolayers were then washed twice with PBS, cells scraped with a rubber policeman and all liquid removed by centrifugation (5′; 3000 rpm; 4 °C) and pellets stored at −80 °C until use. The cell pellets were lysed in 100 μL lysis buffer (50 mM Tris-HCl, pH 7.4, 150 mM NaCl, 1%, NP-40, 1 mM EDTA, 5% glycerol, 1 mM dithiothreitol, 1 mM phenylmethylsulfonyl fluoride, with one tablet of protease inhibitor mixture (Roche Molecular Biochemicals, Basel, Switzerland) per 10 mL of buffer added just prior to use) for 10 min on ice. Equal amounts of lysates were assayed by the Bradford Protein Assay method (Quick Start™ Bradford Protein Assay Kit 1, Bio-Rad Laboratories S.r.l. 20054 Segrate (MI), Italy). Roughly 10–40 μg total protein/lane were loaded and separated through 12% SDS-page, transferred to PVDF membranes using the iBlot dry blotting system (Thermo Fisher Scientific Inc. Monza, MB ITALY), and blotted for the p53 (DO-1, Santa Cruz Biotechnology, Inc. 69115 Heidelberg, Germany), p21 (c-19, Santa Cruz), and b-actin (Sigma Aldrich, Merk Life Science S.r.l. Milano, MI, Italy. Cat A2066, Sigma). The secondary antibodies were anti-mouse HRP conjugate for p53 and p21 and anti-rabbit HRP conjugate for b-actin.

Blot image analysis was performed with UV-Tech Cambridge Imaging System, (Voden Medical Instruments SPA. 20821 Meda, MB, Italy). The signal intensity measured for each band on immunoblots was normalized to the loading control and the fold increase was determined in relation to the appropriate negative control.

Statistical analysis

Data for growth curves and anti-clonal and anti-proliferative assays were obtained from at least three independent experiments. Each data point of quantitative anti-proliferative or anti-clonal experiments represents the mean (±SD) of eight independent replicates. Data were analyzed and plotted using Prism 10.0.1 software (GraphPad Software, San Diego, CA, USA). Interaction analysis and evaluation was performed with the help of Combenefit soft-ware (version 2.021), an interactive, open-source platform for the analysis of combinatorial effects of drugs released by The Cambridge University (UK) [26].

Ethical issues

This research raised no ethical concerns, being entirely based on in vitro experiments involving neither animal use nor patient-derived materials and information. In compliance with National and International ethical rules, no informed consent was needed.

## 3. Results

A literature-based short list of promising polyphenols and flavonoids was selected. Currently, no low-molecular-weight drug is available for clinical use in HPV-related conditions, despite the large body of work that has been conducted in recent years. Polyphenols and flavonoids are a very popular large family of small compounds credited with a wide range of potentially interesting biological activities including antibacterial, anti-inflammatory, antioxidant, and anti-proliferative effects. In the search for new anti-HPV agents, following a critical re-evaluation of literature data and based on anecdotical suggestions, theoretical consideration, and previous structural and biochemical studies, we selected, from a group of roughly 50 promising candidates, a short list of 16 molecules (listed in Table 1) to be further characterized as potential new antineoplastic agents. These molecules were initially screened for their gross anti-clonal activity.

A few RNPs show strong anti-clonal activity.

**Figure 1:**
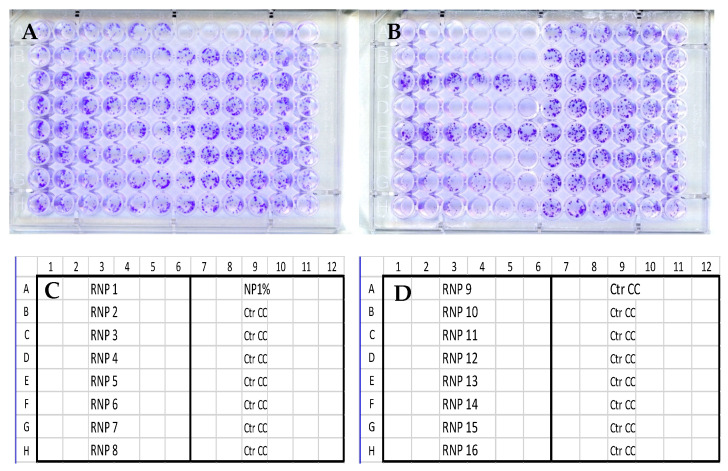
Anti-clonal effects of polyphenols on HeLa cells. Panel (**A**) 50 µM of each RNP 1–8 was administered to cells in rows A–H, columns 1–6 (six replicas per condition). None of compounds induced any noticeable effects as compared with control untreated cells (rows B–H, columns 7–12). An experimental positive control (NP) is shown in row A, wells 7–12. Panel (**B**) 50 µM of each RNP 9–16 was administered to cells in rows A–H, columns 1–6. Clearly, an almost complete suppression of clonal activity is clear with Kaempferol (RNP9), Galangin (RNP10), Luteolin (RNP12), Chrysin (RNP14), Quercetin (RNP15), and Apigenin (RNP16), respectively, in rows A, B, D, F, G, and H, columns 1–6. Conversely, no effect can be seen with RNP11 Pinobanksin and RNP13 Rutin. Panel (**C**,**D**) diagrammatic topographical maps of treatments and growth conditions on plates A and B, respectively. Comparable results were obtained both on SiHa and ME-180 cells.

The end-point anti-clonal effects of the polyphenols on the HeLa cells are shown in Figure 1.

**Figure 2:**
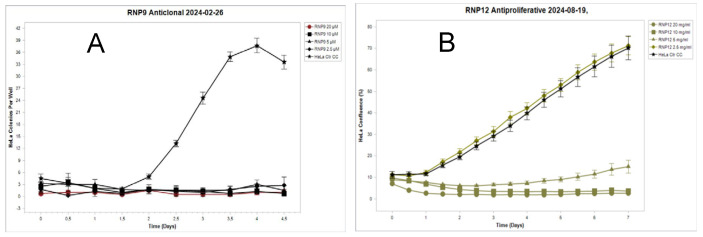
Anti-clonal effect on HeLa cells by live cell image analysis. Panel (**A**) Anti-clonal effect of RNP9. A complete clonal suppressive effect was shown with concentrations down to 2.0 μM for RNP9. Panel (**B**) RNP12 at 5.0 µM induced almost complete suppression of clonal activity.

As can be seen (Panel A), the components (1) Vanillic acid, (2) Caffeic acid, (3) Coumaric acid, (4) Ferulic acid, (5) Benzoic acid, (6) Chlorogenic acid, (7) Protocatechuic acid, and (8) Pinocembrin used isolated were devoid of any significant anti-clonal effect. Conversely (Panel B), the compounds (9) Kaempferol, (10) Galangin, (12) Luteolin, (14) Chrysin, (15) Quercetin, and (16) Apigenin, once administered at 50 μM, each induced almost complete clonal suppression. To confirm and extend this observation, limiting dilution experiments were run to determine their 50% clonal inhibitory dose (CID_50_) both through qualitative and quantitative anti-clonal assays. The results of a representative experiment involving RNP12 Luteolin and RNP9 Kaempferol are reported in Figure 2. Anti-clonal effects of RNPs 10, 14, 15, and 16 are reported in Supplementary Figure S1.

The whole set of RNP CID_50_ values thus obtained are shown in the third (right) column of Table 1. As can be seen, a CID_50_ as low as 1.25 μM could be measured for Kaempferol, of 6.25 μM for Galangin, and of 3.0 μM for Luteolin, while a CID_50_ of 25.0 μM was revealed for Chrysin and Apigenin, and of 40.0 μM for Quercetin. Vanillic acid and Caffeic acid showed a faint anti-clonal effect, with CID_50_ values of 80 and 90 μM, respectively. No other compound showed any anti-clonal activity, although all of them resulted as invariably toxic at concentrations higher than 120–150 μM. However, these values, roughly corresponding to the average concentration of nutrients and amino acids in culture media [27], were regarded as exerting a gross, unspecific toxic effect and were devoid of any pharmacological interest. Almost superimposable results were obtained for each compound with the SiHa and ME180 cells.

A few RNPs had distinct anti-proliferative and anti-clonal activities

**Figure 3:**
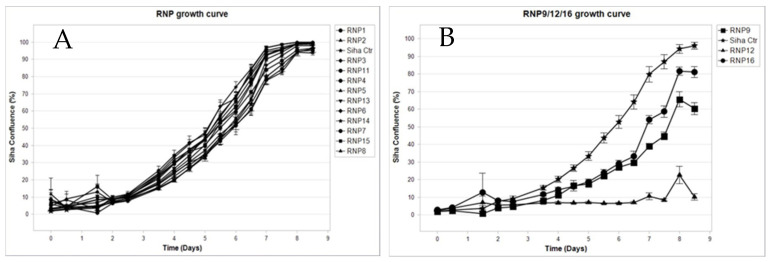
Live cell analysis of RNPs’ anti-proliferative effect on SiHa cells. Panel (**A**) growth curve for RNP1-8 showing no anti-proliferative effect for any molecule. Panel (**B**) growth curve for RNP9 RNP10 and RNP12 showing a fair anti-proliferative effect. Each point represents the mean of six independent replicas. Bar width represents the Standard Deviation (SD).

Interestingly, in addition to the reported anti-clonal effect, a sharp anti-proliferative effect on established tissue cultures could be shown for the compound RNP12 Luteolin and, although to a milder extent, for compounds RNP9 Kaempferol and RNP16 Apigenin as well (Figure 3).

The SiHa cells were seeded in a 96-well plate as described in Section 2, challenged with each RNP at 50 µM, and allowed to grow for up to 196 h (eight days). Images were taken every 12 h; growth curves were drawn plotting the percentage of confluence versus time. Panel A: No anti-proliferative effect can be detected for RNP1 Vanillic acid, RNP2 Caffeic acid, RNP3 Coumaric acid, RNP4 Ferulic acid, RNP5 Benzoic acid, RNP6 Chlorogenic acid, RNP7 Protocatechuic acid, RNP8 Pinocembrin, RNP10 Galangin, RNP11 Pinobanksin, RNP13 Rutin, RNP14 Chrysin, and RNP15 Quercetin. In fact, their growth curves outline a single spindle completely overlapping and masking one of the control untreated cells. Conversely, a distinct anti-proliferative effect is evident in Panel B, where the growth curves of RNP9 Kaempferol (squares), RNP12 Luteolin (diamonds), RNP16 Apigenin (circles), and control untreated cells (stars) are depicted. Luteolin induces almost complete suppression of proliferation while Apigenin and Kaempferol can achieve partial suppression, roughly of 20% and 40%, respectively. Each point represents the mean of six independent replicas. Bar width represents the Standard Deviation (SD). Consistent results were obtained using the HeLa and ME-180 cells.

Cooperative effects of RNP combinations

Polyphenols and flavonoids, because of their common structural features and the vast different substitutions and derivatizations they can undergo, have the potential to interact with, and modulate the activity of, a very wide range of different unrelated cellular regulators [5,8,16,17]. Thus, it is possible that different combinations of polyphenols, through the modulation of an unpredicted pattern of related and unrelated cell pathways, might arouse interesting and unexpected emerging pharmacological properties. To evaluate this possibility, multiple combinations of active RNPs have been assayed for their anti-clonal and anti-proliferative effects.

**Figure 4:**
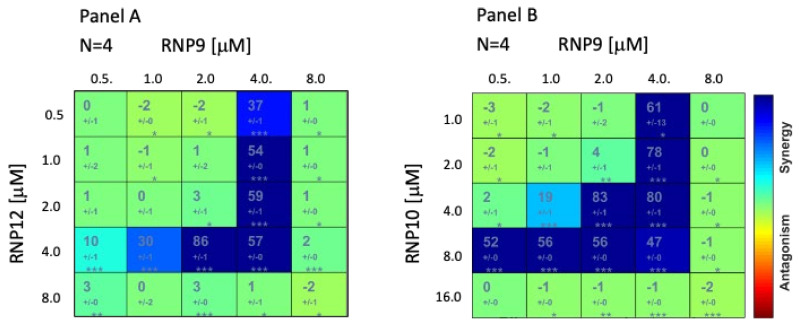
RNP9/12 and RNP9/10 cooperative activity. Matrix display of Bliss Independence model for the RNP9/12 and RNP9/10 combination couples (panel (**A**) and (**B**), respectively). Left upper digit index = replicate assay number; large numerical box index = cooperation score; small numerical box index = SD; *, **, and *** = *p* value < 5 *×* 10^−2^, 10^−3^, and 10^−4^, respectively; right chromatic vertical bar = quality of interaction (synergistic/antagonistic).

Interestingly the three most active compounds, i.e., the RNP9 Kaempferol, RNP10 Galangin, and RNP12 Luteolin turned out to elicit clear cooperative effects at concentrations in the range of their respective CID_50_ values (Figure 4).

**Figure 5:**
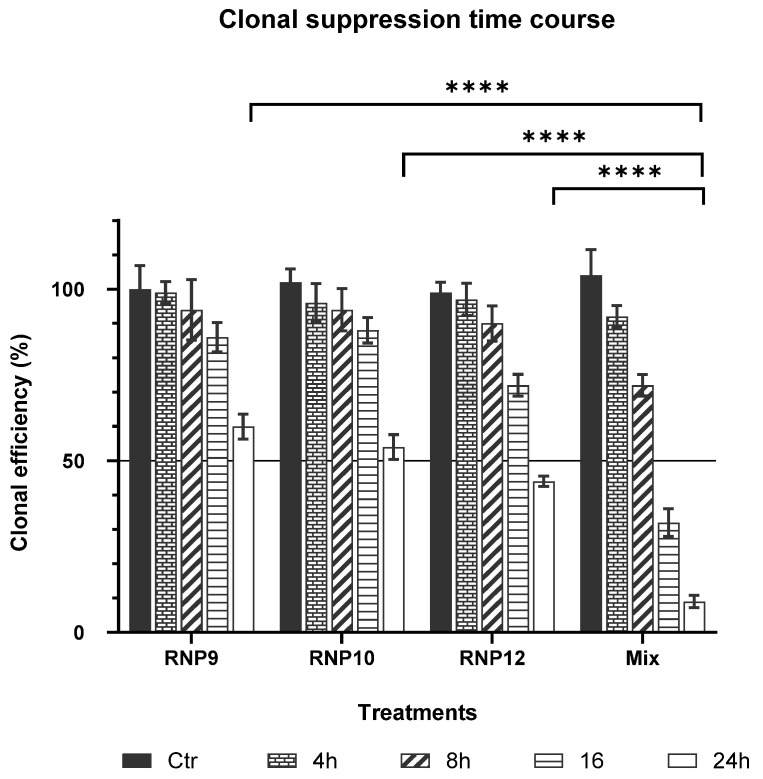
Time-course SiHa clonal suppression of a Kaempferol/Galangin/Luteolin combination. Kaempferol, Galangin, and Luteolin were administered to SiHa cells at the concentrations of 25.0 µM each and in a combination containing 12.5 µM, 12.5 µM, and 6.25 µM, respectively. After 4, 8, 16, and 24 h treatments, cells were washed out and refed with fresh medium and eventually assayed for anti-clonal effects by live cell image analysis. Almost 24 h are needed for any single component to induce roughly 50% clonal inhibition. Conversely, the triple combination readily inhibited the clonal activity after only an 8 h treatment and its extent clearly was much sharper than those of single components after a 24 h long treatment. Thus, the triple combination outperforms any single component by a shorter time of induction and extent of effect. Comparable results were also obtained with HeLa cells. Each value represents the mean ± SD of 8 independent replicas. The solid horizontal line with a “y” value of 50% represents the CID_50_ clonal inhibition threshold. Two-way ANOVA tests for comparisons of multiple groups were performed with GraphPad software, version 10.0. **** = *p* value < 10^−4^.

Based on these stimulating results, the effects of a triple combination made of RNP9 Kaempferol, RNP10 Galangin, and RNP12 Luteolin were examined and are displayed in Figure 5. In these washout experiments, the duration of exposure to induce clonal suppression was evaluated. Clearly, the triple mixture outperforms each single component both in terms of a shorter time of induction and larger extent of clonal suppression (Figure 5).

As can be seen, almost 24 h of exposure to either RNP9 Kaempferol, RNP10 Galangin, or RNP12 Luteolin, as single components, are needed to induce at least 50% inhibition of clonal activity. Conversely, the triple combination, after as little as 16 h, readily induced distinct clonal inhibition, the extent of which largely exceeded the 50% threshold after 24 h of exposure. The triple combination thus outperforms its single components in terms of a shorter time of induction and in terms of the extent of the effect. The parallel experiments with the HeLa cells yielded comparable results. Each value represents the mean ± SD of eight independent replicas. The solid horizontal line with a 50% “y” value marks the CID_50_ clonal inhibition threshold. Statistical analyses were performed with GraphPad software, version 10.0, using a two-way ANOVA for comparisons of multiple groups (* *p* < 0.05, ** *p* < 0.01).

The RNP treatments reduced the cell viability

**Figure 6:**
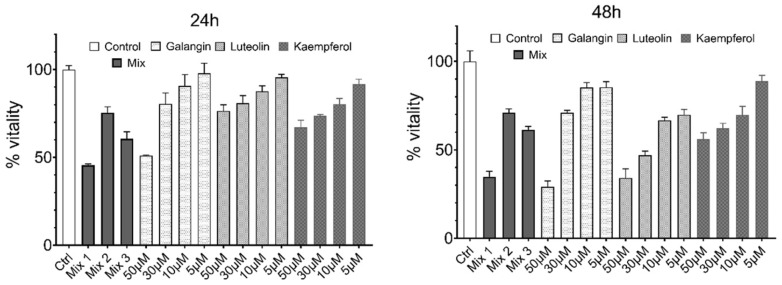
RNP modulation of cell vitality. WST assay on HeLa cells treated for 24 h and 48 h with increasing concentrations (5 μM, 10 μM, 30 μM, and 50 μM) of each compound (RNP9 Kaempferol, RNP10 Galangin, and RNP12 Luteolin) or a mixture of the three components (Mix 1: 10.0 μM RNP9, 10.0 μM RNP10, and 5.0 μM RNP12; Mix 2: RNP9, RNP10, and RNP, 12 5.0 μM each; Mix 3: RNP9, RNP10, and RNP12, 10.0 μM each). DMSO 0.2% *v*/*v*, corresponding to the maximal amount of DMSO present in testing solutions, was added to the medium of control cells. Values are expressed as mean and SEM (*n* = 3).

The effect of the RNPs on the cell viability was evaluated using the WST assay. The cells were treated for 24 h and 48 h with increasing concentrations (5 μM, 10 μM, 30 μM, and 50 μM) of each compound (RNP9 Kaempferol, RNP10 Galangin, and RNP12 Luteolin). Each substance was able to reduce the cell vitality by more than 50% at the highest concentration used (50 μM), with Galangin and Luteolin showing the strongest effect, with 30% and 35% residual vitality at 48 h, respectively (Figure 6). Different combinations of the three components were also tested to reveal potential cooperative effects. As can be seen, Mix 2, including the three components at 5 μM each, reduced the cell viability at 48 h to 70% in the control cells and Mix 3 (each component at 10 μM) reduced the cell viability down to about 60%. These reductions, although moderate, are, however, consistently sharper than those of each single component at the same cumulative concentration, strongly suggesting a cooperative effect of the three substances on cell viability. Interestingly, a triple combination at a ratio of RNP9/RNP10/RNP12 = 10/10/5 μM induced a 45% reduction in the cell viability as early as 24 h post-administration.

The RNPs’ effects are coupled with a relative increase in cells in the G_2_/M phase

**Figure 7:**
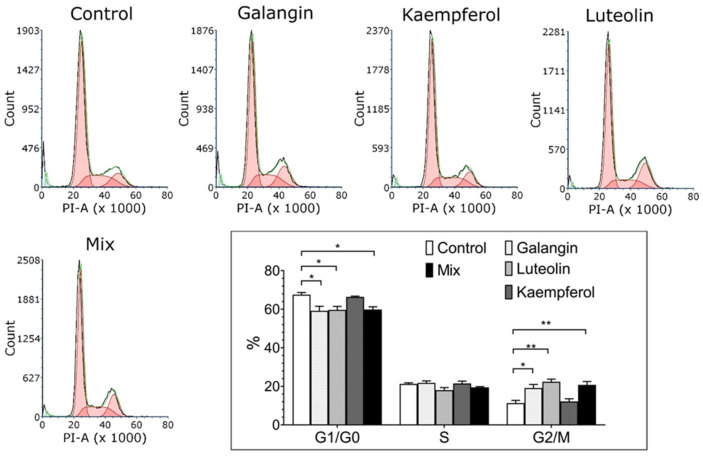
RNP cell cycle modulation. Cell cycle distribution profiles of HeLa cells treated for 16 h with RNP9; RNP10 and RNP12 at 15 μM each and with Mix1 (RNP9 Kaempferol and RNP10 Galangin at 10 μM and RNP12 Luteolin at 5 μM). The black lines refer to experimental data, green lines to fit curves and red area the cell distribution in the different phases. Treated cells show a tendency to reduce S-phase cells and enrich G_2_-phase cells as compared with control untreated cultures. The histogram shown in the right panel summarizes the cell percentage in the different phases. Data were analyzed by FCS Express 7, shown as means and SD, and are representative of three independent measurements. Statistical analyses were performed with GraphPad software, version 10.0.01, using ANOVA followed by Tukey’s post hoc test for comparisons of multiple groups (* *p* < 0.05, ** *p* < 0.01).

A cell cycle analysis was performed on the HeLa cells upon treatment with the RNPs alone and in combinations. Based on the results obtained from the cell vitality assay, the combination of RNP9 Kaempferol and RNP10 Galangin, both at 10 μM, and RNP12 Luteolin at 5 μM was selected because of its maximal cytotoxic effects. The results are shown in Figure 7. As can be seen, after 24 h of treatment, both RNP10 and RNP12 as well as Mix 1 led to a definite increase in cells in the G_2_/M phase, suggesting that the anti-proliferative effects observed could be related to a block in the G_2_/M checkpoint. Surprisingly RNP9, although able to induce both anti-clonal and anti-proliferative effects, at 15 μM, seemed to have no effect on cell cycle progression.

RNP9, RNP10, and RNP12 increase the level of p53 and p21 proteins

**Figure 8:**
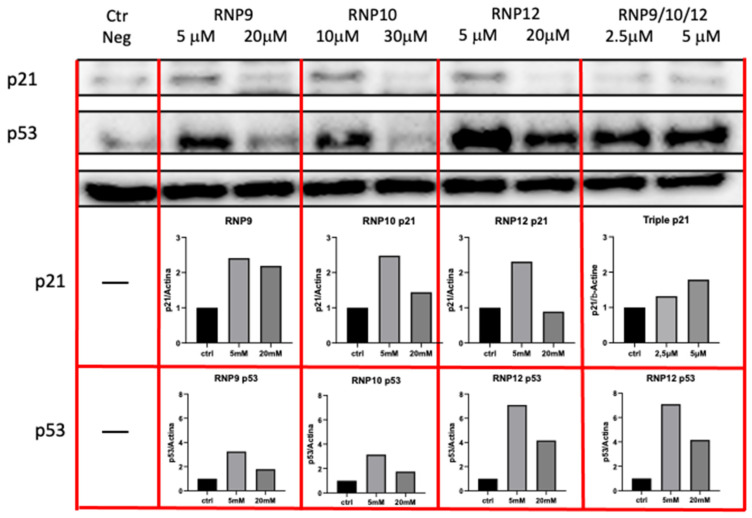
RNP treatment raises the level of the p53 and p21 cellular proteins. SiHa cells (35 × 10^3^/cm^2^) were seeded into a six-well plate and allowed to incubate overnight. Then, monolayers were washed twice and refed with fresh medium containing RNPs at the indicated concentrations. After a further overnight incubation, cells were lysed and the total levels of cellular p21 and p53 were measured by immunoblot as described. Original Western blot images can be found in Figure S3.

Several polyphenols have been shown to have the structural features to interact with the HPV E6 oncogene and disrupt its binding to the cellular protein ligase E6 Associate Protein (E6AP). This interaction is a fundamental step for the suppression of p53’s function, a major mechanism in HPV carcinogenesis. To ascertain whether the molecules used here did actually interact with and disrupt the E6–E6AP binding, and therefore relieve E6’s suppression of p53’s function, we measured, in a Western blot assay, the level of the p53 protein and its downstream effector p21. Both of these two proteins are expected to increase upon the suppression of E6/E6AP binding. The results of a representative experiment are shown in Figure 8. We found that, as compared with the negative control, all three components, administered at 5 μM each, clearly induced an elevation in both p53 and p21 proteins and comparable effects were obtained with the administration of the triple combination.

## 4. Discussion

HPV-related infectious and neoplastic diseases are going to remain a concern for human health for many years to come and new therapeutic options are urgently needed. Currently, no low-molecular-weight molecules are available for clinical use despite the large body of work performed [28,29]. Polyphenols and flavonoids, a sub-class of polyphenols mostly found as metabolic intermediates in vegetable cells, have been described to have a wide range of pleiotropic cellular modulatory effects including antibacterial, anti-inflammatory, antioxidant, immune-modulating, and anti-proliferative activities [16,20,21,30,31].

Based on the large amount of literature data and on previous studies on molecular structure and biochemical characterization [32,33], we decided to evaluate the biological properties of several polyphenols potentially able to interact with HPV oncogenic functions. These molecules are listed in Table 1. Among these sixteen putative anti-HPV drugs, six molecules, namely, RNP9 Kaempferol, RNP10 Galangin, RNP12 Luteolin, RNP14 Chrysin, RNP15 Quercetin, and RNP16 Apigenin, indeed proved able to suppress the clonal activity of HPV-transformed cells, with almost superimposable results shared by SiHa, CaSki, HeLa, and ME180 cells, respectively, transformed by HPV16, HPV18, and HPV68 (see Figure 1 and Figure 2). To evaluate precisely the anti-clonal activity, titration experiments were set up. The results obtained (right column in Table 1) indicate, for all the active molecules, a fair level of effect. Indeed, all of the CID_50_ values are in the micromolar range. Our concentrations are considerably lower than those generally reported as active in most in vitro experimental work conducted with polyphenols [30,31,32,33], which not rarely report concentrations as high as 100 micromolar or above. Indeed, our doses are close to the 1 μM in vitro pharmacological dosage of hydrocortisone and its derivatives, here assumed as a prototype of the ligand/receptor interaction model. As already stated, concentrations over the 100 micromolar threshold are of minimal if any pharmacological interest, being rather in the range of nutrients in culture media [27]. Thus, our results indicate that RNPs efficiently interact with a biologically relevant mechanism(s) is the context of living cells.

Considering that, in our experimental setting, the treatments were administered concomitantly with the cell plating, the reported effects must be strictly considered as evidence of anti-clonal activities suppressing the cells’ ability to attach to a substrate, polarize, and start replication. As a matter of fact, the removal of the treatments 24 h post-seeding did not restore clonal activity (Figure 5). Indeed, no cellular clone was ever revealed by the careful end-point scrutiny of the plating experiments, indicating that the RNP treatments interfered with very early stages of cell adhesion and polarization.

However, with an appropriate experimental setup, it is possible to separate the anti-clonal effect and anti-proliferative effects. This can be easily achieved by administering the pharmacological treatment 24 h post-seeding, when cell adhesion and polarization have been almost completed and cell replication is already launched and ongoing. With such an approach, we were able to show for the three components, RNP9 Kaempferol, RNP12 Luteolin, and RNP16 Apigenin, distinct anti-proliferative activity. Interestingly, this anti-proliferative effect is not shared by other anti-clonal RNPs. Moreover, anti-clonal and anti-proliferative effects did not correlate in their extent, indirectly lending support to the hypothesis that each RNP interacts with more than one cellular target and modulates at least partly distinct pathways. Indeed, it has been shown that polyphenols and flavonoids, because of their common structural features and the vastly different substitutions and derivatizations they can undergo, have the potential to interact, at the biochemical level, with a wide range of different, unrelated cellular targets [27,33]. Thus, it is conceivable that a combination of molecules, through the modulation of several unrelated pathways, might happen to induce unpredicted and favorable effects.

Indeed, several different combinations of RNPs turned out to possess either synergistic or antagonistic effects and, interestingly, among them, a triple combination made of RNP9, RNP10, and RNP12 revealed the better synergistic profile with a shorter time of induction and lower CID_50_ compared with each single component (Figure 4 and Figure 5).

The triple combination, as well as all of its components, is also associated with a significant impact on WST reduction (Figure 6) and, once again, proved to be more active than any single component. Indeed, the triple combination was revealed to possess an anti-metabolic total RNP CID_50_ of 25 μM, while at least 50 μM was needed for a similar effect with RNP9 Galangin or RNP12 Luteolin to induce comparable metabolic suppression. The WST test is usually regarded as a surrogate marker of cell proliferation. As a matter of fact, WST reduction is performed by microsomal dehydrogenases and therefore it must be regarded as a surrogate marker of cell metabolic activity rather than of cell proliferation. Keeping this in mind, our results indicate that, together with the reported anti-clonal and anti-proliferative effects, the RNP treatments induced distinct suppression of cell metabolic activity, the extent of which does not mirror the anti-clonal activity, as is shown by RNP9, which has only a scant anti-metabolic effect (Figure 5) despite its distinct anti-clonal activity. The fact that anti-clonal, anti-proliferative, and anti-metabolic effects can be, at least partially, uncoupled through appropriate experimental settings, lends support to the hypothesis that each RNP may interact and modulate at least partially distinct sets of cellular pathways.

The above reported results are further associated with the induction of a clear reduction in cells in the S-phase compartment of the cell cycle and a parallel accumulation of cells in the G_2_/M compartment, consistent with a (partial) restoration of the G_2_/M checkpoint. As a whole, the above results indicate that RNP treatments are able to induce global suppression of cell activity and that different pathways can be differentially modulated.

HPV-transformed cells continuously express the three viral oncogenes E5, E6, and E7, which provide the biochemical conditions required for enhanced responsivity to EGF and survival signals, the suppression of apoptosis and surveillance of DNA replication, and unrestricted cell cycle progression. Three powerful conditions fueling the cell neoplastic progression. The reported suppression of global cell activity is consistent with the hypothesis of the reduced activity of viral oncogenes. Many polyphenols have been proven to specifically interact with the E6 oncogene and disrupt its interaction with E6AP, the initiating step in the functional suppression of p53 [32,33,34]. Thus, we asked whether such an interaction actually takes place and eventually generates measurable effects within the intact cell. Regrettably, E6 function cannot be easily evaluated because of the lack of convenient commercial reagents and robust specific antibodies. Thus, we evaluated the cellular level of p53 and its downstream effector p21 as two surrogate markers of E6 activity in the hypothesis that the suppression of E6 function, no matter how it was brought about, would suppress the proteasome degradation of the p53 protein, leading to its own elevation and that of the downstream p53-dependent CDK inhibitor p21. Indeed, this is exactly what we showed in the Western blot experiment, where, once again, the triple combination of RNP9, RNP10, and RNP12 proved to perform better than any of the isolated components.

## 5. Conclusions

According to the reported data, it was surprisingly found that the well-known polyphenolic compounds RNP9 Kaempferol, RNP10 Galangin, and RNP12 Luteolin display strong anti-clonal and anti-proliferative activity on cells transformed by HPV16, HPV18, or HPV68. A similar anti-clonal effect, although to a milder extent, is also shared by RNP14 Chrysin, RNP15 Quercetin, and RNP16 Apigenin.

This effect is coupled with parallel, distinct anti-proliferative and anti-metabolic effects and with a partial arrest of the cell cycle at the G_2_/M checkpoint.

The association of different RNPs resulted either in synergistic or antagonistic effects and a triple combination of RNP9, RNP10, and RNP12 consistently proved more active than any single component, suggesting that each RNP holds the potential to activate, to different extents, multiple cellular pathways. The identification of a synergistic drug combination is a major goal in the search for new pharmacological tools. Nonetheless, this fascinating concept still lacks a sound and unanimously shared definition [26,35,36,37]. Accordingly, a number of different mathematical approaches have been developed. Each of them provide crucial although partial answers and all of them are affected by different pros and cons (stringently reviewed in Duarte D & Vale N. 2022 [36]). Entering the debate on the use of these statistical tools for biomedical research is out of the scope of this work. However, in wishing to provide a quantitative assessment of our results and for the sake of data reusability, we evaluated the reported data with three different approaches that are very popular and commonly used in pre-clinical research. Namely, the Highest Single Agent (HSA), the Bliss independence model, and the Loewe Additivity model. These three models are not equivalent; rather, they often yield different and non-overlapping results depending on the experimental conditions and settings. As can be seen in Figure 4 and in Figure S2, comparable, mostly superimposable results were recorded, meaning our findings were consistent with cooperative effects. To reflect this conceptual landscape, throughout this manuscript, we avoided using the term *synergy* and conservatively adopted the more colloquial descriptive definition of *cooperative effect*.

These cooperative effects are associated with a sharp restoration of the p53 protein levels and, consistently, with the consequent elevation of the CDK inhibitor p21, suggesting, in accordance with literature data, that the RNPs brought about the specific disruption of the E6–E6AP interaction, the crucial step in oncogene-mediated p53 suppression.

In any case, these effects were achieved at a pharmacological (micromolar) concentration and the triple RNP combination consistently turned out to be more active than each individual component.

However, at a preliminary level, these results point to the possible development of oncogene-suppressing low-molecular-weight treatments.

## 6. Limitations

This study reports initial results and, accordingly, provides a proof of principle for the potential effect of RNP 9, RNP10, and RNP12 and their cooperative combination on HPV-transformed cell lines. The reported results need to be extended to a wider range of transformed cells, including primary keratinocytes transformed with isolated HPV oncogenes expressed by lentiviral vectors. Moreover, the analysis of HPV oncogenic mechanisms should be extended at least to the E5- and E7-dependent pathways. These are indeed our immediate experimental plans; nonetheless, they are demanding and time-consuming and thus fall out of the scope of this first communication.

## Figures and Tables

**Table 1:** Polyphenols and Flavonoids.

Molecule		CID_50_ (µM)
RNP1	Vanillic acid	80.00
RNP2	Caffeic acid	90.00
RNP3	Coumaric acid	>120.00
RNP4	Ferulic acid	>120.00
RNP5	Benzoic acid	>120.00
RNP6	Chlorogenic acid	>120.00
RNP7	Protocatechuic acid	>150.00
RNP8	Pinocembrin	>150.00
RNP9	Kaempferol	1.25
RNP10	Galangin	6.25
RNP11	Pinobanksin	>150.00
RNP12	Luteolin	3.00
RNP13	Rutin	>150.00
RNP14	Chrysin	25.00
RNP15	Quercetin	40.00
RNP16	Apigenin	25.00

## Data Availability

The data reported here are covered by the Italian Industrial Invention patent no. 102022000016647, property of NP1 Srl Roma, Italy. The raw data for this manuscript are available at the following link: (https://gbox.garr.it/garrbox/f/650103871, accessed on 17 February 2025). The data presented in this study are available on request from the corresponding author due to legal and commercial restrictions.

## Supplementary Materials

The following supporting information can be downloaded at https://www.mdpi.com/article/10.3390/pathogens14030221/s1: Figure S1: Anti-clonal effect of Live cell analysis of RNPs 10; 14; 15 and 16; Figure S2: Loewe and HAS RNP9/12 and RNP9/10 cooperative activity; Figure S3: Original WB images for p53 and p21 proteins level.
