# Supplementary material for: A Combination of Flavonoids Suppresses Cell Proliferation and the E6 Oncogenic Pathway in Human Papillomavirus-Transformed Cells
Source: Pathogens. 2025 Feb 24;14(3):221. doi: 10.3390/pathogens14030221 (PMC11945172; doi:10.3390/pathogens14030221)
Supplement: Supplementary file 1 [file pathogens-14-00221-s001.zip › pathogens-3417151-supplementary.pdf]

Panel A

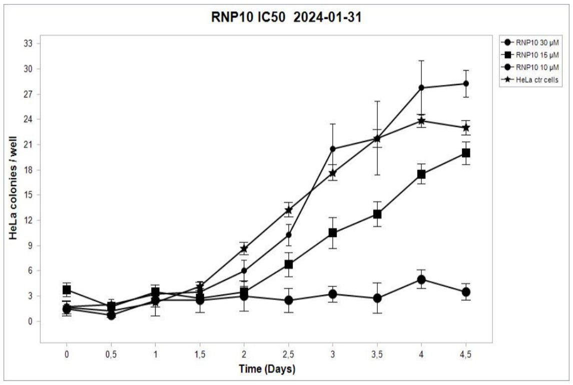

Panel B

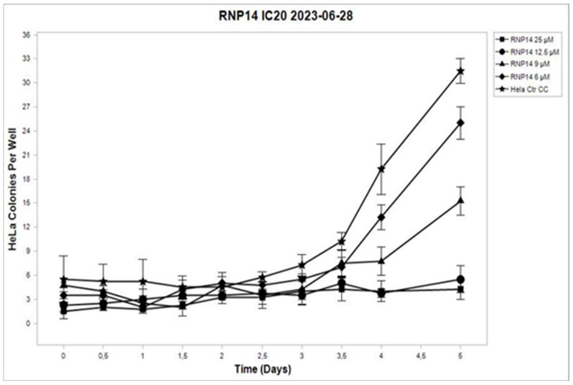

Panel C

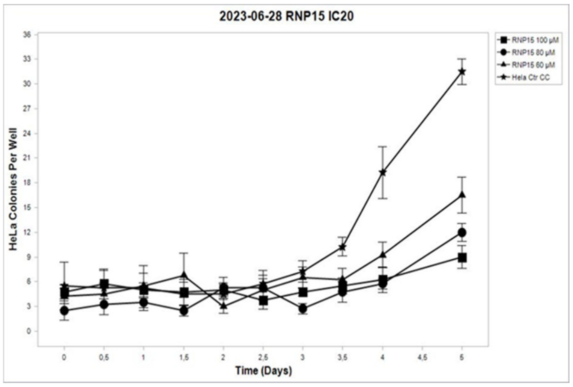

Panel D

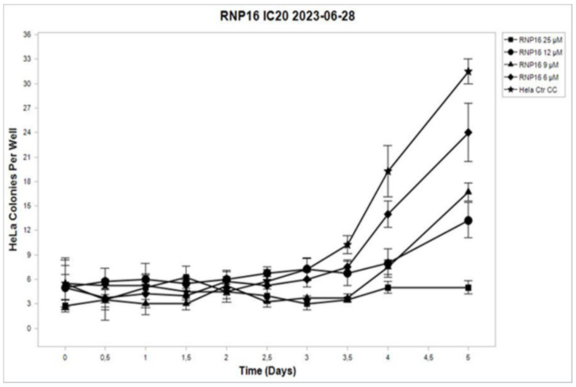

Supplementary figure s1. - Anti-clonal effect of Live cell analysis of RNPs 10; 14; 15 and 16.

HeLa cells were seeded in a 96 well plate and assayed for clonal efficiency and for proliferative activity in presence of RNP10 Galangin; RNP14 Chrysin; RNP15 Quercetin and RNP16 Apigenin-1; in the INCUCYTE S3 Imaging System. The following CID_{50} could be determined: 15 µM for RNP10 Galangin (panel A); 9 µM for RNP14 Chrysin (panel B); 60 µM for RNP15 Quercetin (panel C) and 9 µM for RNP16 Apigenin-1 (panel D). Each value represents the mean ± SD of 8 independent replicas. Almost superimposable results were obtained both with SiHa and ME180 cells.

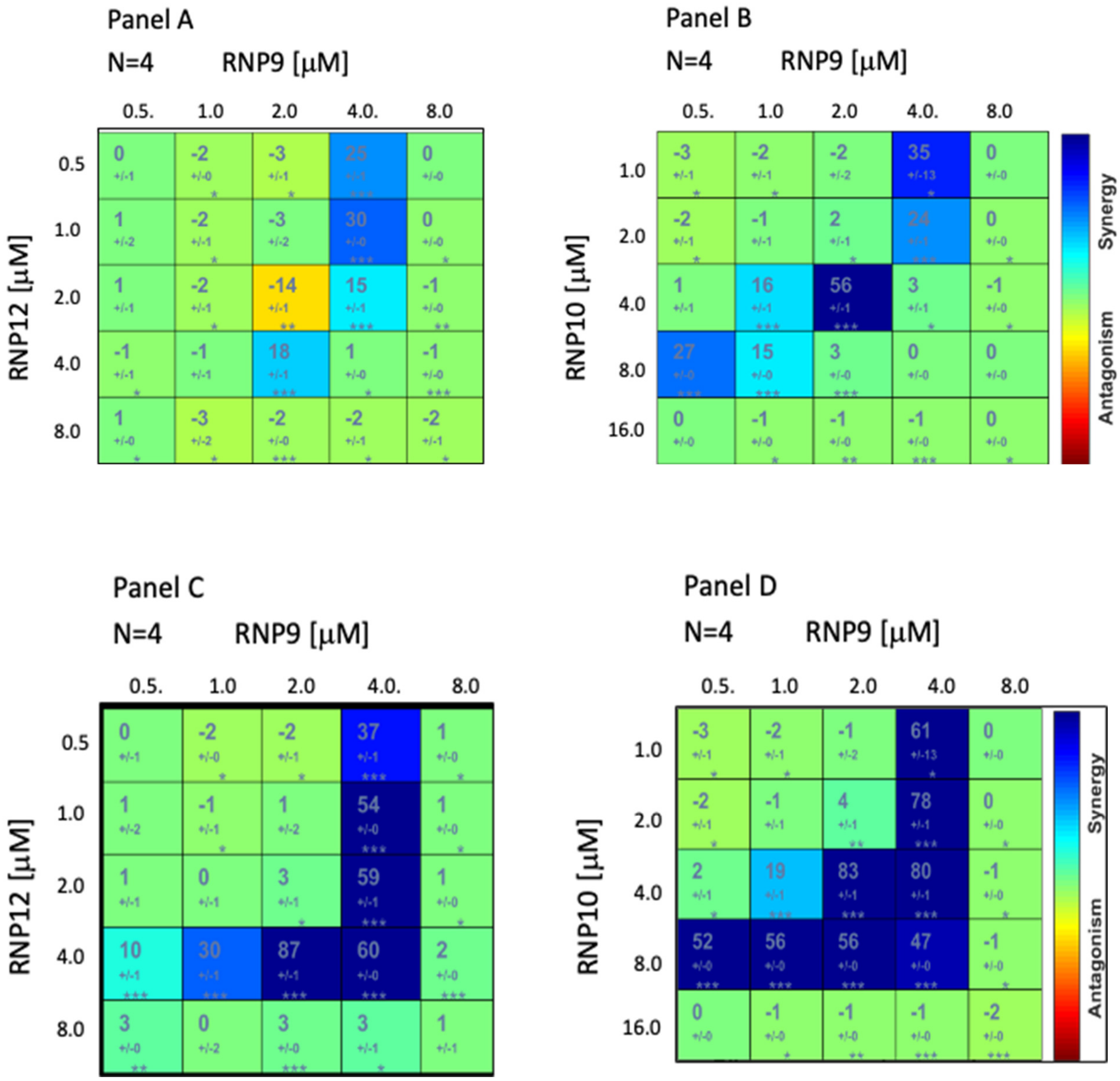

Supplementary figure S2. Loewe and HAS RNP9/12 and RNP9/10 cooperative activity.

Matrix display of Loewe Additivity (panels A & B) and HSA (Highest Single Agent) interaction (panel C & D). Left upper index = replicate assay number; large numerical box index = Cooperation score; small numerical box index = SD; *, ** and *** = p value $<5\times10^{-2}$; $<10^{-3}$ and $<10^{-4}$ respectively; Right chromatic vertical bar = quality of interaction (synergistic/antagonistic). Di Veroli,G.Y. et al. (2016) [26]. (http://bioinformatics.oxfordjournals.org/content/early/2016/05/27/bioinformatics.btw230.abstract) https://sourceforge.net/projects/combenefit/files/Combenefit%202.02%20WIN_64%20%28PREFERRED%29/ (accessed on 22 December 2024).

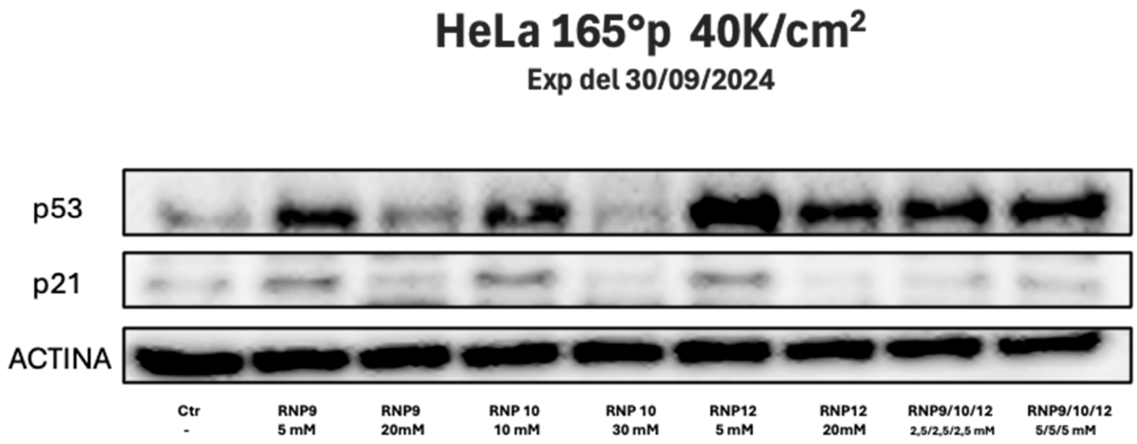

Figure S3. Original WB images for p53 and p21 proteins level.

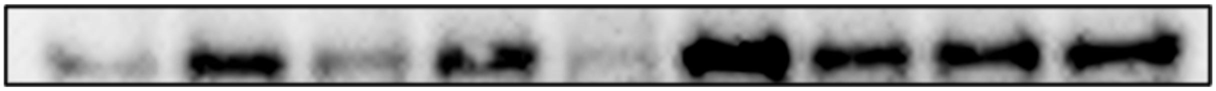

Sub-Figure S3a. Original WB images for p53.

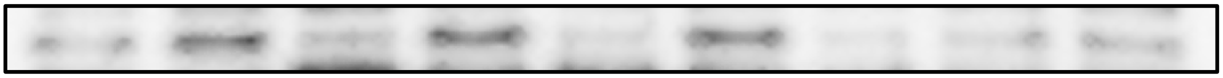

Sub-Figure S3b. Original WB images for p21.

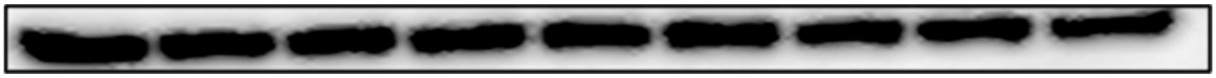

Sub-Figure S3c. Original WB images for β-actin.

The raw data for uncropped, unedited WB analyses of p53 and p21 proteins are available at the following link: https://gbox.garr.it/garrbox/f/650103871 (accessed on 22 December 2024).
